# Hypercalcemia as a Presenting Clinical Manifestation of Adenocarcinoma of the Colon

**DOI:** 10.4021/wjon512w

**Published:** 2012-08-26

**Authors:** Angelo Argento

**Affiliations:** Internist, Valley View Regional Hospital, 2020 Arlington, Suite 2, Ada, OK 74820, USA. Email: argento99@medscape.com

**Keywords:** Hypercalcemia, Colon cancer, PTH-rp, Prognosis

## Abstract

Hypercalcemia is rarely associated with colon cancer. It is related to overexpression of parathyroid hormone-related protein (PTH-rp) in malignant cells of the primary colon tumor and metastases. A 44 year old lady presented for evaluation of severe hypercalcemia (15.7 mg/dL) associated with abdominal pain, nausea and constipation. She was diagnosed with metastatic colon cancer involving the liver. Therapy for hypercalcemia consisted of intravenous bisphosphonate and saline hydration. Hypercalcemia remained resistant and refractory to treatment despite resection of the colon tumor. She died soon after admission to hospice. It is proposed that malignant cells of the primary colon tumor and distant metastases, in this patient, were the site of ectopic PTH-rp secretion resulting in hypercalcemia. This case illustrates the significance of recognizing hypercalcemia as a potential clue in detecting underlying colon cancer involving overproduction of PTH-rp. It also exemplifies the poor prognosis expected with this type of humoral hypercalcemia of malignancy and the difficulty encountered when trying to achieve normalization of calcium in this setting.

## Introduction

Hypercalcemia is commonly associated with solid tumors seen in lung, breast and kidney cancer in addition to hematologic malignancy such as multiple myeloma, leukemia and lymphoma [1]. In contrast, carcinoma of the colon is rarely associated with hypercalcemia which usually implicates a poor prognosis and survival rate [2]. The cell types most often discovered, during histological examination of colon cancer with hypercalcemia, are adenocarcinoma and adenosquamous carcinoma [3-7]. Hypercalcemia presenting as a manifestation of solid tumors is typically due to production of a humoral factor or hormone, by malignant cells, and rarely from local osteolytic destruction secondary to bony metastases. Humoral hypercalcemia of malignancy (HHM) is secondary to overproduction of a polypeptide, belonging to the parathyroid hormone (PTH) family, known as parathyroid hormone-related protein (PTH-rp) [8, 9]. This entity, described as paraneoplastic syndrome, is characterized by ectopic hormone production in the setting of underlying malignancy. Clinical awareness of hypercalcemia as a diagnostic clue and accompanying manifestation leading to detection of underlying colon cancer has become increasingly important because of its prognostic implication and refractoriness to limited available treatment options aimed at normalizing calcium.

## Case Report

A 44-year-old African American female with history of peptic ulcer disease, mitral valve prolapse and anemia presented to the clinic for evaluation of epigastric abdominal pain, nausea and constipation. Review of systems was positive for weight loss of approximately 15 to 20 lbs and fatigue; negative for fever, hematochezia or melena. Medication consisted of dicyclomine, metoclopramide, sucralfate and ferrous sulfate recently prescribed by ER physician for abdominal symptoms. Past surgical history significant for total abdominal hysterectomy with bilateral salpingo-oophorectomy. Family history negative for colon cancer and her mother died from lung cancer at age 70. Physical examination revealed an obese female with blood pressue of 163/88 mmHg and epigastric tenderness to palpation. There were no palpable masses or lymphadenopathy detected. Stool guaic was negative. Laboratory investigation showed calcium level of 15.7 mg/dL, phosphorous 2.8 mg/dL, sodium 136 meq/L, potassium 3.5 meq/L, chloride 100 meq/L, bicarbonate 31 meq/L, BUN 30 mg/dL, creatinine 1.9 mg/dL, glucose 121 mg/dL, total protein 8.1 gm/dL, albumin 3.6 gm/dL, total bilirubin 0.4 mg/dL, SGOT 75 U/L, SGPT 50 U/L and alkaline phosphatase 227 U/L. White blood cell count was 11.3 K/mm^3^, HgB 8.6 gm/L, HCT 27.2 %, MCV 75.6 fL and platelet count 778 K/mm^3^, CEA 702 ng/mL. Intact PTH was not measured. Instead, the carboxy (C)-terminal PTH assay (includes C-terminal, intact PTH and midmolecule) was measured and found to be within normal limits at 48 ng/dL (reference range < 88 ng/dL; Specialty Laboratories, Valencia, CA).

**Figure 1:**
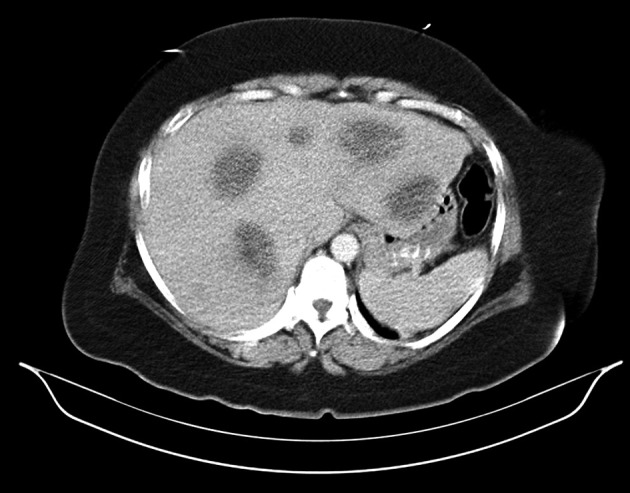
CT scan of the abdomen and pelvis with and without contrast illustrating multiple liver lesions, largest measuring 4 to 5 cm.

**Figure 2:**
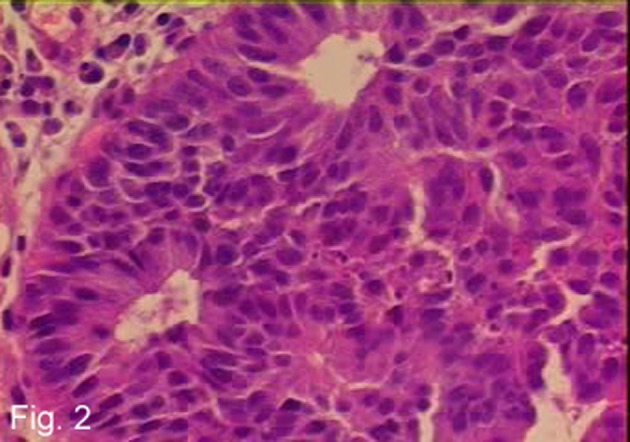
H and E stain of primary colon tumor showing poorly differentiated adenocarcinoma of the colon.

She was admitted for evaluation and treatment of severe hypercalcemia. After administering IV pamidronate (Aredia) along with normal saline and IV furosemide, calcium level gradually declined from 15.7 to 10.1 mg/dL over a period of 7 days. CXR was normal and screening mammogram revealed BIRAD 2 benign findings. CT scan of abdomen with and without contrast showed multiple lesions within the liver (Fig. 1). Colonoscopy revealed a 3 cm ulcerated mass near the splenic flexure (biopsy showed moderately differentiated adenocarcinoma). EGD was normal. The following day after surgical consultation she was taken to the operating room for exploratory laparotomy undergoing segmental resection of the transverse colon, where the identified cancer was located (Fig. 2). Open core needle biopsy of a liver mass demonstrated adenocarcinoma compatible with metastasis from colon.

She was admitted on two additional occasions about 1 week apart, 6 days after initial hospital discharge, for refractory hypercalcemia associated with nausea, vomiting, lethargy and confusion. Temporary and partial response was observed to IV pamidronate, in addition to saline diuresis and IV furosemide, lowering calcium from 15.6 to 12.8 mg/dL over period of 10 days. On the second occasion, hypercalcemia was partially corrected with IV zoledronic acid (Zometa), along with saline diuresis and furosemide, dropping calcium from 16.2 to 13.1 mg/dL over 3 days. After being admitted to hospice, her condition continued to deteriorate and she died 7 weeks after initial hospital admission.

## Discussion

Hypercalcemia, commonly found in malignancy, is a very rare clinical manifestation of colon cancer, most frequently in association with poorly differentiated adenocarcinoma, demonstrated in Figure 2, and adenosquamous carcinoma of the colon [2-7]. Symptoms of hypercalcemia include nausea, vomiting, constipation, abdominal pain, confusion and fatigue most of which were experienced by this patient.

HHM associated with colon cancer has been shown to be mediated by overexpression of PTH-rp in tumor cells [9]. Normal cells also produce PTH-rp which plays a physiological role in skeletal development, epithelial cell growth, smooth muscle relaxation and keratinocyte differentiation [10]. The amino (N)-terminal of PTH-rp shares partial amino acid sequence and tertiary conformational homology with PTH, enabling it to bind to the PTH receptor leading to increased bone resorption and distal tubular calcium reabsorption along with decreased proximal tubular reabsorption of phosphate; ultimately resulting in hypercalcemia and hypophosphatemia [11]. Patients with hypercalcemia and colon cancer have elevated serum PTH-rp levels with low normal to suppressed PTH in most cases; in addition to, positive staining for PTH-rp in neoplastic cells of both the primary colon tumor and liver metastases [3-7]. Serum PTH-rp was not measured and immunohistochemical staining for PTH-rp was not performed in this patient. Although intact PTH was not measured in this patient, the C-terminal PTH which includes intact PTH was within normal range, supporting the presence of ectopic PTH-rp as the cause of hypercalcemia in this case.

Colon cancer in the presence of hypercalcemia indicates advanced stage disease with liver being the most common site of distant metastases followed by lung and bone. Skeletal metastasis from colon cancer is very infrequent, occurring in approximately 5-10% of patients. Retrospective studies have demonstrated a temporal pattern in which liver and, more accurately, lung precedes bone metastasis in all cases of diagnosed colon cancer [12, 13]. It is unlikely, in this patient, that hypercalcemia was due to local osteolytic tumor invasion in the absence of lung metastases which, is more precise than liver metastases at predicting, and in most cases precedes bony metastasis in patients with colon cancer.

Hypercalcemia, when present, in the setting of underlying colon cancer is a poor prognostic indicator with survival time ranging from 6 days to 7 months. Median survival has been shown to be approximately 4 weeks with mean survival of 8 weeks; one reported case of patient still alive with disease at 13 months [3-6]. HHM associated with colon cancer is often refractory and resistant to treatment, usually recurring within 2 - 3 weeks after therapy is discontinued [14]. Available treatment options for correcting hypercalcemia in these patients are limited and include calcitonin, IV bisphosphonates such as pamidronate and zoledronic acid, along with saline hydration and furosemide. Calcitonin is used in combination with saline hydration for the initial management of severe hypercalcemia (> 14 mg/dL) because it lowers serum calcium concentration rapidly by a maximum of 1 to 2 mg/dL beginning within 4 - 6 hours. Bisphosphonates are more potent and preferred agents, in conjunction with calcitonin and saline, for managing malignancy-related hypercalcemia with maximum effect occurring in 2 - 4 days [15, 16]. Zoledronic acid is more powerful and sometimes favored over pamidronate because it can be administered over a shorter period, 15 minutes compared to 2 hours [17].

Although PTH-rp was not measured and immunohistochemical staining for PTH-rp was not performed in this case, a normal level of C-terminal PTH which includes intact PTH in association with hypercalcemia, suggests the presence of a humoral factor or hormone, namely PTH-rp. It is believed with high likelihood that malignant cells of the primary colon tumor and liver metastases, in this patient, were the focus of ectopic PTH-rp production and secretion leading to humoral hypercalcemia of malignancy. This case emphasizes the importance of recognizing hypercalcemia as a presenting manifestation and potential clinical diagnostic clue in detecting underlying colon carcinoma as part of a paraneoplastic syndrome involving overproduction of PTH-rp.
